# Cost-effectiveness of Screening for Osteoporosis in Older Men With a History of Falls

**DOI:** 10.1001/jamanetworkopen.2020.27584

**Published:** 2020-12-01

**Authors:** Kouta Ito

**Affiliations:** 1 Division of Geriatric Medicine, University of Massachusetts Medical School, Worcester; 2 Meyers Primary Care Institute, Worcester, Massachusetts

## Abstract

**Importance:**

Falls and osteoporosis share the potential clinical end point of fractures among older patients. To date, few fall prevention guidelines incorporate screening for osteoporosis to reduce fall-related fractures.

**Objective:**

To assess the cost-effectiveness of screening for osteoporosis using dual-energy x-ray absorptiometry (DXA) followed by osteoporosis treatment in older men with a history of falls.

**Design, Setting, and Participants:**

In this economic evaluation, a Markov model was developed to simulate the incidence of major osteoporotic fractures in a hypothetical cohort of community-dwelling men aged 65 years who had fallen at least once in the past year. Data sources included literature published from January 1, 1946, to July 31, 2020. The model adopted a societal perspective, a lifetime horizon, a 1-year cycle length, and a discount rate of 3% per year for both health benefits and costs. The analysis was designed and conducted from October 1, 2019, to September 30, 2020.

**Interventions:**

Screening with DXA followed by treatment for men diagnosed with osteoporosis compared with usual care.

**Main Outcomes and Measures:**

Incremental cost-effectiveness ratio (ICER), measured by cost per quality-adjusted life-year (QALY) gained.

**Results:**

Among the hypothetical cohort of men aged 65 years, the screening strategy had an ICER of $33 169/QALY gained and was preferred over usual care at the willingness-to-pay threshold of $100 000/QALY gained. The number needed to screen to prevent 1 hip fracture was 1876; to prevent 1 major osteoporotic fracture, 746. The screening strategy would become more effective and less costly than usual care for men 77 years and older. The ICER for the screening strategy did not substantially change across a wide range of assumptions tested in all other deterministic sensitivity analyses. At a willingness-to-pay threshold of $50 000/QALY gained, screening was cost-effective in 56.0% of simulations; at $100 000/QALY gained, 90.8% of simulations; and at $200 000/QALY gained, 99.6% of simulations.

**Conclusions and Relevance:**

These findings suggest that for older men who have fallen at least once in the past year, screening with DXA followed by treatment for those diagnosed with osteoporosis is a cost-effective use of resources. Fall history could be a useful cue to trigger assessment for osteoporosis in men.

## Key Points

**Question:**

Is it cost-effective to screen for osteoporosis in older men with a history of falls?

**Findings:**

In this model-based economic evaluation of a hypothetical cohort of men aged 65 years, screening with dual-energy x-ray absorptiometry followed by treatment for those diagnosed with osteoporosis had an incremental cost-effectiveness ratio of $33 169 per quality-adjusted life-year gained and was preferred over usual care at the willingness-to-pay threshold of $100 000 per quality-adjusted life-year gained.

**Meaning:**

These findings suggest that, for older men who have fallen at least once in the past year, screening with dual-energy x-ray absorptiometry followed by treatment for those diagnosed with osteoporosis is a cost-effective use of resources.

## Teaser

This economic evaluation assesses the cost-effectiveness of screening for osteoporosis using dual-energy x-ray absorptiometry followed by osteoporosis treatment in a hypothetical sample of older men with a history of falls.

## Introduction

Falls and osteoporosis are geriatric syndromes that share the potential clinical end point of fractures.^1^ Approximately 1 in 4 men 65 years or older fall each year in the United States, making falls a public health concern, particularly among older adults.^2^ Prevalence of osteoporosis also increases with age, affecting an estimated total of 2 million men.^3^ By 2030, the number of hip fractures in men is projected to increase to 109 000 each year.^4^ Hip fractures are overwhelmingly precipitated by falls.^5^ Many older men at risk for falls are predisposed to fractures by undetected osteoporosis.^6,7,8,9^ It therefore makes clinical sense to screen for osteoporosis as a part of routine fall prevention care. Performing dual-energy x-ray absorptiometry (DXA) in those who have fallen could be crucial because a history of falls does not necessarily indicate osteoporosis and a need for osteoporosis treatment. To date, few fall prevention guidelines incorporate screening for osteoporosis to reduce fall-related fractures.^10,11,12^ Although several professional organizations have advocated routine screening for osteoporosis in older men, the US Preventive Services Task Force continues to conclude that evidence was insufficient to justify this practice.^13,14,15,16^ Medicare coverage for DXA is rather restrictive in men unless they have known skeletal abnormalities or secondary causes of osteoporosis.^17^ Despite a predicted rise in the number of hip fractures, osteoporosis remains underdiagnosed and undertreated in men.^18^ Recent clinical trials suggested that population-based screening for osteoporosis could reduce hip and osteoporotic fractures in older women.^19,20,21,22^ A similar clinical trial would be ideal to evaluate the efficacy of a screening practice in older men but unrealistic because of the need for a larger sample size. Accordingly, we developed a simulation model to evaluate the long-term health and economic effect of screening with DXA followed by treatment for those diagnosed with osteoporosis in older men with a history of falls.

## Methods

### Overview and Model Structure

**Figure 1.**
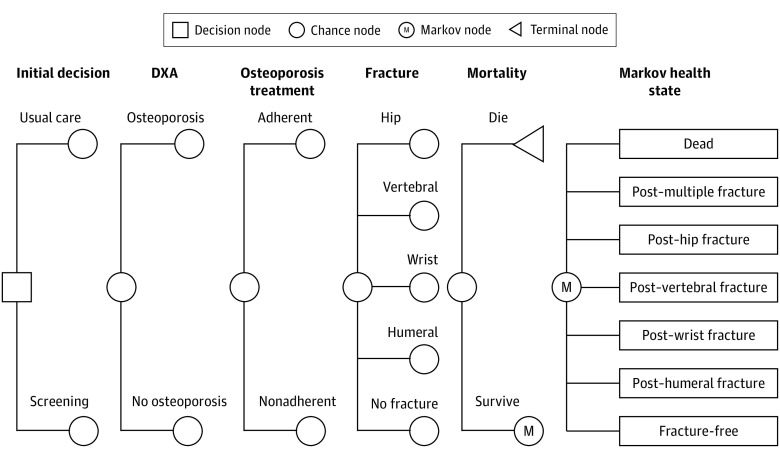
Markov Model Structure All men entered the model in the fracture-free state. At the decision node, they were assigned to either usual care or screening. They had osteoporosis or no osteoporosis at baseline. Osteoporosis treatment was provided only if they were diagnosed with osteoporosis by screening and adherent to recommended therapy. Each year, they were at risk for sustaining a fracture or dying of other causes. If they sustained hip or clinical vertebral fractures, they were at risk of dying due to those fractures. Depending on an event experienced, they remained in the fracture-free state, proceeded to the postfracture states, or were absorbed into the dead state. In the actual model, the post–multiple fracture state was separated into 15 health states representing any combinations of different types of fractures. DXA indicates dual-energy x-ray absorptiometry.

For this economic evaluation study, a Markov model was developed to simulate the prognosis of older men with a history of falls in the last year based on previously published models of screening for osteoporosis and the guideline by the European Society for Clinical and Economic Aspects of Osteoporosis, Osteoarthritis, and Musculoskeletal Diseases and the US branch of the International Osteoporosis Foundation (Figure 1).^23,24,25,26,27^ Health states were determined by a history of major osteoporotic fractures to represent those who experienced any combination of different types of fractures. Data sources included literature published from January 1, 1946, to July 31, 2020. The model adopted a societal perspective, a lifetime horizon, a 1-year cycle length, and a discount rate of 3% per year for both health benefits and costs.^28^ The analysis was performed by using TreeAge Pro Suite 2018 software, version 18.2.1-v20180828 (TreeAge Software). This study follows the recommendations of the Consolidated Health Economic Evaluation Reporting Standards (CHEERS) reporting guideline.^29^

### Population

The model targeted a hypothetical cohort of US community-dwelling men aged 65 years who had fallen at least once in the past year. It was assumed that all men were fracture free at baseline.

### Interventions

Men were assigned to either usual care or screening for osteoporosis. Those who were assigned to usual care underwent no screening for osteoporosis. Those who were assigned to screening for osteoporosis underwent DXA of the femoral neck and the lumbar spine and received treatment if they were diagnosed with osteoporosis. Osteoporosis was defined as a T score of bone mineral density (BMD) of less than −2.5 at either site using the reference for White women aged 20 to 29 years from the National Nutrition and Examination Survey (NHANES) III.^30^ An alternate scenario using the reference for White men aged 20 to 29 years was also tested in a scenario analysis. Alendronate sodium (70 mg orally once a week for 5 years) was selected as a drug of choice when osteoporosis treatment was indicated. An alternate scenario selecting zoledronic acid (5 mg intravenously once a year for 5 years) as a drug of choice was also tested in a scenario analysis. We assumed that a basic metabolic panel was ordered and comprehensive oral examination was performed before initiation of zoledronic acid therapy. Those receiving osteoporosis treatment were assumed to require an additional physician visit each year during a 5-year course of therapy and to incur the cost of DXA in the second and fourth year after treatment initiation.^23,26,31^ We also assumed that both groups were offered the same fall prevention measures and received a daily supplementation of calcium (1200 mg) and vitamin D (800 IU).

### Input Parameters

Model parameters are summarized in Table 1 and eTable in the Supplement and described in detail below. Prevalence of osteoporosis, incidence of fractures, and utility for a fracture-free state were age dependent. Other parameters were considered as constant for each age group.

**Table 1.** Model Parameters

Parameter	Value (range)	Distribution	Source
Discount rate, %	3 (0-6)	Not applied	Neumann et al^28^ (2016)
Prevalence of osteoporosis, %^a^	3.3 (1.98-4.62)	Beta	Wright et al^3^ (2014)
Relative risk of hip fractures associated with a history of falls			
Base case	1.54 (1.21-1.95)	Log-normal	Leslie et al^8^ (2019) and Harvey et al^9^ (2018)
1 Fall^b^	1.51 (1.06-2.15)		
2 Falls^b^	1.88 (1.12-3.16)		
≥3 Falls^b^	3.41 (2.19-5.31)		
Relative risk of nonhip fractures associated with history of falls			
Base case	1.51 (1.32-1.73)	Log-normal	Leslie et al^8^ (2019) and Harvey et al^9^ (2018)
1 Fall^b^	1.44 (1.23-1.67)		
2 Falls^b^	1.65 (1.31-2.08)		
≥3 Falls^b^	2.52 (2.05-3.11)		
Relative risk of fractures associated with the presence of osteoporosis			
Hip	5.98 (3.50-9.06)	Log-normal	Looker et al^32^ (2012) and Johnell et al^33^ (2005)
Nonhip	2.51 (2.01-3.12)		
Relative risk of fractures associated with a history of prior fractures			
Hip	1.97 (1.12-3.48)	Log-normal	Kanis et al^34^ (2004)
Nonhip	1.91 (1.50-2.43)		
Relative risk of fractures during osteoporosis treatment			
Clinical vertebral	0.4 (0.11-1.51)	Log-normal	Nayak and Greenspan^35^ (2017)
Nonvertebral	0.6 (0.40-0.90)		
Treatment benefit offset, y	5 (0-10)	Not applied	Hiligsmann et al^27^ (2019)
Adherence to osteoporosis treatment, %			
Alendronate	43 (32-54)	Beta	Kothawala et al^36^ (2007) and Koller et al^37^ (2020)
Zoledronic acid^b^	36 (23-50)		
Relative risk of death associated with a history of falls	1.3 (0.90-1.80)	Log-normal	Dunn et al^38^ (1992)
Relative risk of death after hip fractures			
First year	3.7 (3.31-4.14)	Log-normal	Haentjens et al^39^ (2010)
Subsequent years	2.53 (1.81-3.54)		
Relative risk of death after clinical vertebral fractures	1.83 (1.80-1.86)	Log-normal	Lau et al^40^ (2008)
Cost, $^c^			
DXA	39.99	Gamma	American College of Rheumatology^41^ (2020)
Physician visit	76.06		American College of Rheumatology^41^ (2020)
Osteoporosis treatment			
Alendronate	250	Gamma	Drugs for postmenopausal osteoporosis^42^ (2020)
Zoledronic acid^b^	515	Gamma	Drugs for postmenopausal osteoporosis^42^ (2020), Insinga^43^ (2016), CMS^44^ (2020), California Department of Health Care Services^45^ (2015)
Fracture event			
Hip	31 713	Gamma	Gabriel et al^46^ (2002) and Kilgore et al^47^ (2013)
Clinical vertebral	9656		
Wrist	8804		
Humeral	5237		
Post–hip fracture state (per year)	11 736	Gamma	Schousboe et al^23^ (2007)
Utility multiplier			
Fracture free^a^	0.84 (0.80-0.85)	Log-normal	Hanmer et al^48^ (2006) and
History of falls	0.97 (0.94-0.98)		Theim et al^49^ (2014)
Fracture event			
Hip	0.55 (0.53-0.57)	Log-normal	Svedbom et al^50^ (2018) and Lesnyak et al^51^ (2020)
Clinical vertebral	0.68 (0.65-0.70)		
Wrist	0.83 (0.82-0.84)		
Humeral	0.76 (0.72-0.79)		
Postfracture state			
Hip	0.86 (0.84-0.90)	Log-normal	Svedbom et al^50^ (2018) and Lesnyak et al^51^ (2020)
Clinical vertebral	0.85 (0.82-0.87)		
Wrist	0.99 (0.97-1.00)		
Humeral	0.89 (0.85-0.92)		

Abbreviations: CMS, Centers for Medicare & Medicaid Services; DXA, dual-energy x-ray absorptiometry.

^a^ Age-specific variable. See the eTable in the Supplement.

^b^ Tested in a scenario analysis.

^c^ Range is 50% to 200% of the base-case costs.

#### Prevalence of Osteoporosis

The age-dependent prevalence of osteoporosis was based on a cross-sectional study from the 2005-2010 NHANES.^3^ The cohort studies from 3 countries^6,7,8^ did not observe a significant difference in BMD among men with and without a history of falls. Therefore, we assumed that the prevalence of osteoporosis was not affected by a history of falls or the number of falls in the previous year. The base-case estimate of the prevalence of osteoporosis was altered widely in sensitivity analyses.

#### Incidence of Fractures

The age-dependent fracture rates were obtained from hospital discharge data from the Healthcare Cost and Utilization Project Nationwide Inpatient Sample.^52,53^ The model modified the fracture rates based on a history of falls, the presence of osteoporosis, and a history of fractures. The relative risks of fractures associated with a history of falls were taken from a meta-analysis of cohort studies from 3 countries.^9^ Pooled data from men with a history of a single fall and multiple falls were obtained and were assumed to last as long as 10 years. An alternate scenario considering the number of falls was also tested in a scenario analysis. The relative risks of fractures stratified by the number of falls (ie, 1, 2, or ≥3) in the past year were based on the combined data for men and women from a cohort study in Canada.^8^ The relative risks of fractures associated with the presence of osteoporosis were calculated using BMD data from the 2005-2008 NHANES and a meta-analysis of cohort studies from 12 countries.^32,33^ The excess risks of fractures associated with a history of fractures were taken from a meta-analysis of cohort studies from 11 countries.^34^

#### Treatment Effect

Relative risk reductions of fractures by osteoporosis treatment were taken from a meta-analysis of clinical trials of bisphosphonates in men.^35^ We assumed that a history of falls did not alter the fracture reduction benefit by osteoporosis treatment. The model used the reduction in nonvertebral fracture rates as a surrogate for the reduction in hip, wrist, and humeral fracture rates.^27^ The fracture reduction benefit was assumed to appear in the second year of therapy.^54^ The model incorporated a linear decrease in the fracture reduction benefit during 5 years after its termination.^27^ Adherence to alendronate and zoledronic acid treatment was based on meta-analyses of multiple observational studies.^36,37^ The model adopted a conservative assumption that only those who completed a 5-year course of osteoporosis treatment gained the fracture reduction benefit. Adverse effects of treatment were not modeled based on a systematic review.^16^

#### Mortality

The background mortality rates were based on 2017 US life tables published by the National Center for Health and Statistics.^55^ Excess mortality attributable to a history of falls was taken from the Longitudinal Study on Aging.^38^ Excess mortality after hip fractures was taken from a meta-analysis of cohort studies from 7 countries.^39^ Excess mortality after clinical vertebral fractures was taken from a retrospective data analysis of Medicare claims.^40^ An alternate scenario excluding excess mortality after clinical vertebral fractures was also tested in a scenario analysis.^27^

#### Costs

The costs of DXA and a physician visit were obtained from the 2020 Medicare National Average Rates (*Current Procedural Terminology* [*CPT*] codes 77080 and 99213, respectively).^41^ The wholesale acquisition costs of alendronate and zoledronic acid were used as the medication costs.^42^ Those who did not adhere to alendronate therapy accrued the medication cost for only 6 months. Those who did not adhere to zoledronic acid therapy accrued the medication cost for only the first year. Infusion and related supply costs of zoledronic acid were taken from a US claim-based cost analysis.^43^ Costs of the basic metabolic panel (*CPT* code 80048) and comprehensive dental examination (*CPT* code D 0150) were based on the national mean commercial rates.^44,45^ Acute costs of managing each type of fracture were taken from a US claim-based cost analyses.^46,47^ The long-term cost of hip fractures (eg, increased admission to long-term care facilities) was incorporated.^23^ All costs were inflated to 2019 US dollars using the Consumer Price Index for Medical Care for All Urban Consumers.^56^

#### Quality of Life

The age-dependent, fracture-free utility values were based on EuroQol-5 Dimension (EQ-5D) scores collected from nationally representative US community-dwelling samples.^48^ The utility multiplier for a history of falls was calculated using a EQ-5D survey in Germany.^49^ The utility multiplier for each type of fracture was taken from the International Costs and Utilities Related to Osteoporotic Fractures Study.^50,51^ If individuals sustained a fracture, for example, their baseline utilities were multiplied by the utility of that fracture event in the first year and by the utility of that postfracture health state in subsequent years.

### Model Validation

To assess its external validity, the model simulated the prognosis of men aged 50 years without a history of falls receiving usual care. The predicted lifetime risk of hip fracture was 11%; clinical vertebral fracture, 9%; wrist fracture, 3%; and humeral fracture, 3%. These predicted risks approximated well with published estimates.^57^

### Statistical Analysis

The analysis was designed and conducted from October 1, 2019, to September 30, 2020. We measured health outcomes in quality-adjusted life-years (QALYs). The incremental cost-effectiveness ratio (ICER) of a strategy was calculated as the additional cost of that strategy divided by its additional health benefit compared with the competing strategy. Deterministic sensitivity analyses were conducted on discount rates, age at screening, treatment characteristics (ie, treatment effect during treatment and after discontinuation), medication treatment adherence, excess mortality after fractures, treatment costs, fracture costs, and effect of fractures on utility. The 95% CI of each parameter was obtained, when available; otherwise, 50% to 200% of the base-case estimates were used. Because the reduction in nonvertebral fractures with treatment was used as a surrogate for reduction in hip fracture rates, this assumption was tested rigorously in sensitivity analyses. A probabilistic sensitivity analysis was also conducted in which the model was run using a value for each parameter down randomly from the distribution assigned to that parameter. The model ran 100 000 iterations to generate a cost-effectiveness acceptability curve showing the probability that either strategy was cost-effective using varying willingness-to-pay (WTP) thresholds.

## Results

### Base-Case Analysis

For the base-case population, 1876 men needed to undergo screening to prevent 1 hip fracture, and 746 men needed to undergo screening to prevent 1 major osteoporotic fracture (Table 2). The screening strategy improved quality-adjusted survival by 0.0026 QALYs, increased costs by $87, and had an ICER of $33 169/QALY gained. Therefore, the screening strategy would be preferred over usual care at the conventional WTP threshold of $100 000/QALY gained.

**Table 2.** Cost-effectiveness According to Age at Screening

Strategy	Number needed to screen		Cost, $	QALYs	ICER, $/QALY
	Hip fracture	MOF			
Aged 65 y (base case)					
Usual care	NA	NA	6447	9.6146	Reference
Screening	1876	746	6534	9.6173	33 169
Aged 70 y					
Usual care	NA	NA	7396	7.8295	Reference
Screening	739	393	7451	7.8339	12 631
Aged 75 y					
Screening	NA	NA	8061	6.1953	Reference
Usual care	482	309	8094	6.2002	6670
Aged 80 y					
Usual care	NA	NA	9017	4.6041	Dominated
Screening	183	104	8932	4.6152	Reference

Abbreviations: ICER, incremental cost-effectiveness ratio; MOF, major osteoporotic fracture; NA, not applicable; QALY, quality-adjusted life-year.

### Sensitivity Analyses

These results were sensitive to assumptions about the age at screening (Table 2). As the target population became older, numbers needed to screen to prevent 1 hip fracture and 1 major osteoporotic fracture would become smaller and the screening strategy would become increasingly cost-effective. The screening strategy would become dominant (ie, more effective and less costly than usual care) for men 77 years or older. These results were also sensitive to the assumption about the treatment effect during osteoporosis treatment. Under the most conservative assumption about the relative risk of nonvertebral fractures during osteoporosis treatment (ie, 0.90), the ICER for the screening strategy would slightly exceed the conventional WTP threshold of $100 000/QALY gained. The screening strategy would be preferred over usual care for men 66 years or older. The ICER for the screening strategy did not substantially change across a reasonable range of assumptions tested in all other deterministic sensitivity analyses (Table 3).

**Table 3.** One-Way Deterministic Sensitivity Analyses

Parameter	ICER, $/QALY	Range
Discount rate, %	19 477-51 655	0-6
Prevalence of osteoporosis, %	21 461-64 966	1.98-4.62
Relative risk of fracture on osteoporosis treatment		
Clinical vertebral	28 034-80 949	0.11-1.51
Nonvertebral	16 765-104 340	0.40-0.90
Treatment benefit offset, y	22 013-67 222	0-10
Adherence to osteoporosis treatment, %	23 806-48 967	32-54
Relative risk of death after hip fracture		
First year	33 053-33 277	3.31-4.14
Subsequent years	32 184-34 421	1.81-3.54
Relative risk of death after vertebral fracture	33 085-33 253	1.80-1.86
Cost, $		
Osteoporosis treatment	29 557-40 392	50%-200% of the base-case costs
Fracture event		
Hip	25 986-36 760	
Clinical vertebral	31 791-33 857	
Wrist	32 911-33 298	
Humeral	32 768-33 369	
Post–hip fracture health state (per year)	20 208-39 649	
Utility multiplier		
Fracture event		
Hip	33 019-33 320	0.53-0.57
Clinical vertebral	33 027-33 263	0.65-0.70
Wrist	33 157-33 181	0.82-0.84
Humeral	33 067-33 245	0.72-0.79
Postfracture health state		
Hip	32 686-34 178	0.84-0.90
Clinical vertebral	32 369-33 581	0.82-0.87
Wrist	33 066-33 220	0.97-1.00
Humeral	32 756-33 485	0.85-0.92

Abbreviations: ICER, incremental cost-effectiveness ratio; QALY, quality-adjusted life year.

### Probabilistic Sensitivity Analysis

**Figure 2.**
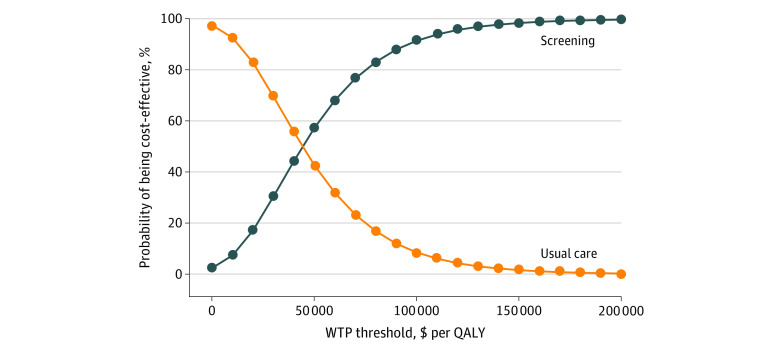
Cost-effectiveness Acceptability Curve A range of willingness-to-pay (WTP) thresholds were plotted on the horizontal axis against the probability that either usual care or the screening strategy would be cost-effective at that WTP threshold on the vertical axis. QALY indicates quality-adjusted life-year.

The result of the probability sensitivity analysis in the base-case population is displayed in the cost-effectiveness acceptability curve (Figure 2). At the WTP threshold of $50 000/QALY gained, the screening strategy would be cost-effective in 56.0% of stimulations; at $100 000/QALY gained, 90.8% of simulations; and at $200 000/QALY gained, 99.6% of simulations.

### Scenario Analyses

If the reference for White men aged 20 to 29 years was applied for the diagnosis of osteoporosis, an ICER for the screening strategy would decrease to $31 039/QALY gained. If zoledronic acid was selected as a drug of choice when osteoporosis treatment was indicated, an ICER for the screening strategy would increase to $52 653/QALY gained. If men with a history of a single fall in the past year were targeted, an ICER for the screening strategy would increase to $34 705/QALY gained. If men with a history of multiple falls in the past year were targeted, an ICER for the screening strategy would decrease to $25 159/QALY gained for men with a history of 2 falls and $5478/QALY gained for men with a history of at least 3 falls. If excess mortality after clinical vertebral fractures was excluded from the model, an ICER for the screening strategy would increase to $36 360/QALY gained. Therefore, across various scenarios tested, the screening strategy would remain preferred over usual care at the conventional WTP threshold of $100 000/QALY gained.

## Discussion

This study found that screening with DXA followed by treatment for those diagnosed with osteoporosis would be reasonably cost-effective for men aged 65 years who have fallen at least once in the past year. If the target population was older than 77 years, it would simultaneously improve health outcomes and save money from the societal perspective. These findings were robust to wide variations of the model assumptions and concur with previous cost-effectiveness analyses indicating that screening could be justified when targeted to men at higher risk for osteoporotic fractures.^23,24,25,26^ These findings are also promising given the emerging evidence that population-based screening using fracture risk assessment and DXA is effective to reduce hip and osteoporotic fractures in postmenopausal women.^19,20,21,22^ The observed numbers needed to screen to prevent a hip and an osteoporotic fracture (ie, 272 and 247, respectively) from these clinical trials are comparable with our model estimates for men aged 75 to 80 years.^22^

Determining whether the patient has fallen in the past year is the first step in preventing future falls and the major injuries that can result from falling.^58^ Fall risk assessment is integrated into the Welcome to Medicare examination and the Medicare Annual Wellness Visit.^59^ In a recent review undertaken by the US Preventive Services Task Force, multifactorial interventions or single exercise-based interventions were found to reduce fall risk, but their effect on fracture risk was not significant.^10^ Fracture prevention could be achieved by screening and treatment for osteoporosis without reducing the number of falls.^60^ These findings do not suggest that fall prevention measures should not be included in patient management but do raise a concern that, for individuals with significant skeletal risk factors, fall prevention measures alone might not be sufficient to reduce fall-related fractures. On the other hand, combined with older age, the presence of osteoporosis accounts for only one-third of all fracture cases in men.^61^ The BMD-based approach is likely to miss a large number of individuals who do not have osteoporosis and have nonskeletal risk factors (eg, falls) and is unlikely to reduce a large number of fractures. A combined approach to address both falls and osteoporosis is not a new concept.^62^ Because fracture prevention is the ultimate goal, inclusion of screening for osteoporosis into the fall prevention algorithm could potentially lead to a further reduction of fractures in individuals who have fallen.^63^

### Limitations

The study should be interpreted in light of several cautions. First, we assumed that the fracture reduction efficacy by osteoporosis treatment was not altered by a history of falls. In a clinical trial of clodronate in postmenopausal women,^64^ fall risk did not significantly affect its fracture reduction efficacy. In a clinical trial of zoledronic acid in institutionalized, functionally impaired women with osteoporosis,^65,66^ improvements in BMD were comparable to those observed in community-dwelling, functionally unimpaired women. None of the clinical trials of bisphosphonates in men were adjusted for their underlying fall risk.^35^ Therefore, we varied the assumption about treatment effectiveness widely in sensitivity analyses. Second, the characterization of risk factors for fracture that contribute significantly to fracture risk, beyond that provided by BMD, has stimulated the development of risk assessment tools to determine a treatment threshold. The more adequately evaluated tools include the Fracture Risk Assessment Tool (FRAX) tool (University of Sheffield),^67^ Garvan bone fracture risk calculator (Garvan Institute of Medical Research),^68^ and the QFracture risk calculator (ClinRisk Ltd).^69^ Both the Garvan and QFracture tools include a history of falls, whereas the FRAX tool does not. Recognizing the limitations of falls data in the current FRAX cohorts, an expert panel recommended that FRAX probability might be modified to account for a history of falls, with the output inflated by 30% for each past fall (for ≤5 falls).^70^ In this study, the model did not consider these risk assessment tools to determine a treatment threshold because drug efficacy in preventing fractures has been proven only in individuals with osteoporosis defined based on BMD or fracture history.

## Conclusions

The results of this economic evaluation suggest that for older men who have fallen at least once in the past year, screening with DXA followed by treatment for those diagnosed with osteoporosis may be a cost-effective use of resources. In clinical practice, fall history could be a useful cue to trigger assessment for osteoporosis in men. The study also suggests that Medicare coverage of screening with DXA could be expanded to older men with a history of falls.

## Notes

Supplement. **eTable.** Age-Dependent Model Parameters
