# Supplementary material for: Cost-effectiveness of Screening for Osteoporosis in Older Men With a History of Falls
Source: JAMA Netw Open. 2020 Dec 1;3(12):e2027584. doi: 10.1001/jamanetworkopen.2020.27584 (PMC7708999; doi:10.1001/jamanetworkopen.2020.27584)
Supplement: Supplement. — eTable. Age-Dependent Model Parameters [file jamanetwopen-e2027584-s001.pdf]

Supplementary Online Content

Ito K. Cost-effectiveness of screening for osteoporosis in older men with a history of falls. *JAMA Netw Open*. 2020;3(12):e2027584. doi:10.1001/jamanetworkopen.2020.27584

eTable. Age-Dependent Model Parameters

This supplementary material has been provided by the authors to give readers additional information about their work.

©2020 Ito K. *JAMA Network Open*.

eTable. Age-Dependent Model Parameters			
**Parameter**	**Value**	**Range**	**Reference**
Prevalence of osteoporosis (%)			3
50-59	3.40	1.94-4.86	
60-69 (base case)	3.30	1.98-4.62	
70-79	5.00	3.75-6.25	
80+	10.90	8.18-16.35	
Incidence of fractures (% per year)		50%-200%	52, 53
Hip			
50-54	0.028		
55-59	0.038		
60-64	0.066		
65-69 (base case)	0.118		
70-74	0.210		
75-79	0.402		
80-84	0.813		
85+	1.630		
Clinical vertebral			
50-54	0.043		
55-59	0.046		
60-64	0.178		
65-69 (base case)	0.114		
70-74	0.214		
75-79	0.350		
80-84	0.358		
85+	1.239		
Wrist			
50-54	0.147		
55-59	0.064		
60-64	0.141		
65-69 (base case)	0.095		
70-74	0.064		
75-79	0.045		
80-84	0.149		
85+	0.094		
Humeral			
50-54	0.027		
55-59	0.048		
60-64	0.081		
65-69 (base)	0.142		
70-74	0.160		
75-79	0.134		
80-84	0.075		
85+	0.188		
Utility multiplier			48
Fracture-free health state			
50-59	0.86	0.85-0.86	
60-69 (base case)	0.84	0.83-0.85	
70-79	0.80	0.79-0.82	
80+	0.78	0.76-0.81	

©2020 Ito K. *JAMA Network Open*.
